# The burden of chronic obstructive pulmonary disease and its attributable risk factors in the Middle East and North Africa region, 1990–2019

**DOI:** 10.1186/s12931-022-02242-z

**Published:** 2022-11-19

**Authors:** Hamidreza Feizi, Mahasti Alizadeh, Seyed Aria Nejadghaderi, Maryam Noori, Mark J. M. Sullman, Javad Ahmadian Heris, Ali-Asghar Kolahi, Gary S. Collins, Saeid Safiri

**Affiliations:** 1 Social Determinants of Health Research Center, Department of Community Medicine, Faculty of Medicine, Tabriz University of Medical Sciences, Tabriz, Iran; 2 Student Research Committee, Tabriz University of Medical Sciences, Tabriz, Iran; 3 Research Center for Integrative Medicine in Aging, Aging Research Institute, Tabriz University of Medical Sciences, Tabriz, Iran; 4 Systematic Review and Meta-Analysis Expert Group (SRMEG), Universal Scientific Education and Research Network (USERN), Tehran, Iran; 5 Student Research Committee, School of Medicine, Iran University of Medical Sciences, Tehran, Iran; 6 Department of Life and Health Sciences, University of Nicosia, Nicosia, Cyprus; 7 Department of Social Sciences, University of Nicosia, Nicosia, Cyprus; 8 Department of Allergy and Clinical Immunology, Pediatric Hospital, Tabriz University of Medical Sciences, Tabriz, Iran; 9 Social Determinants of Health Research Center, Shahid Beheshti University of Medical Sciences, Tehran, Iran; 10 Centre for Statistics in Medicine, NDORMS, Botnar Research Centre, University of Oxford, Oxford, UK; 11 NIHR Oxford Biomedical Research Centre, Oxford University Hospitals NHS Foundation Trust, Oxford, UK

**Keywords:** Chronic obstructive pulmonary disease, Risk factor, Epidemiology, Global burden of disease, Socio-demographic index, Middle East and North Africa

## Abstract

**Background:**

Chronic obstructive pulmonary disease (COPD) is a preventable and treatable disease that is characterised by persistent respiratory symptoms and airflow limitation. The present study reported the burden of COPD, and its attributable risk factors, in the Middle East and North Africa (MENA) region between 1990 and 2019, by age, sex and socio-demographic index (SDI).

**Methods:**

Data from the Global Burden of Disease (GBD) 2019 study were used to report the burden of COPD in the MENA countries. The prevalence, deaths, and disability-adjusted life-years (DALYs) were presented as counts and age-standardised rates per 100,000 population, with their associated 95% uncertainty intervals (UIs).

**Results:**

In 2019, the regional age-standardised point prevalence and rates of death due to COPD were 2333.9 (2230.1, 2443.6) and 26.1 (22.2, 29.5) per 100,000, which represent a 30.6% (28.2%, 33.0%) increase and an 18.0% (2.8%, 30.9%) decrease, respectively, since 1990. The regional age-standardised DALY rate in 2019 was 649.1 (574.6, 717.7) per 100,000, which had decreased by 11.8% (0.9%, 21.1%) since 1990. Turkey had the highest age-standardised point prevalence in 2019 [3287.1 (3187.4, 3380.3)], while Afghanistan had the highest age-standardised death [40.4 (24.2, 52.6)] and DALY [964.5 (681.8, 1203.2)] rates. The regional age-standardised point prevalence, death and DALY rates in 2019 increased with advancing age and were higher in males in almost all age groups. There was a U-shaped association between SDI and the burden of COPD over the period 1990 to 2019. Moreover, in 2019 smoking (43.7%), ambient particulate matter pollution (22.8%) and occupational particulate matter (11.4%) had the largest proportion of attributable DALYs for both sexes.

**Conclusions:**

COPD is one of the leading causes of death and disability in the MENA region, although the age-standardised burden has decreased over the last 30 years. Nevertheless, COPD accounted for a substantial number of deaths and DALYs, especially among the elderly. Programs targeting risk factors, like smoking, should be taken into consideration.

**Supplementary Information:**

The online version contains supplementary material available at 10.1186/s12931-022-02242-z.

## Introduction

Chronic obstructive pulmonary disease (COPD) is defined by the Global Initiative for Chronic Obstructive Lung Disease (GOLD) as “a common, preventable and treatable disease that is characterised by persistent respiratory symptoms and airflow limitation that is due to airway and/or alveolar abnormalities usually caused by significant exposure to noxious particles or gases” [1]. The chief constituents of COPD are emphysema which is a permanent destructive enlargement of airspaces, and chronic bronchitis which is an excretion of sputum for at least 3 months per year for 2 successive years [1, 2]. Ageing, occupational exposure to dust and fumes, indoor and outdoor air pollutants, genetic risk factors and especially tobacco smoking are some of the risk factors for developing COPD [2, 3]. In the primary stages of the disease, the physical symptoms include a chronic cough, excessive sputum excretion, wheezing and shortness of breath and as the disease develops, dyspnea becomes more common [4]. Mental conditions, such as anxiety and depression, are also common comorbidities of COPD and these predict a diminished quality of life, decreased exercise capacity and higher rates of exacerbations, along with increased mortality [5].

COPD has a considerable economic burden, such that in 2011 the yearly direct (i.e. healthcare) and indirect (i.e. production loss) cost of COPD in Europe was 48.4 billion euros [6]. The economic costs of COPD are mostly due to the hospitalization and medication costs and are strongly influenced by the stage of the disease, as well as the severity and frequency of acute exacerbations [7, 8]. COPD is one of the most common causes of mortality and morbidity, and has the highest death rate of all chronic respiratory diseases globally [9]. According to the latest information from the Global Burden of Diseases (GBD) 2019, COPD attributable DALYs ranked 11th in 1990 and 6th in 2019, although the age-standardised DALY rate per 100,000 population decrease by 39.8% during this time period [10]. It has been estimated that COPD affected more than 380 million people globally in 2010, with a higher prevalence in males [11]. Furthermore, the age-standardised prevalence rate of COPD increases with advancing age and in the future is expected to become equal among males and females, mostly due to changes occurring in the smoking habits of females [3].

The deaths and disability-adjusted life-years (DALYs) of COPD have previously been reported using data provided by GBD 2017 [12]. In another study, the burden of COPD was assessed at the super-regional level in conjugation with other chronic respiratory diseases [9]. Moreover, a recent investigation, using data from GBD 2019, only reported the burden of COPD attributable to ambient particulate matter 2.5 [13]. In addition, the burden of COPD at the global, regional and national levels was recently reported [14], but this study did not focus on the Middle East and North Africa (MENA) region. Furthermore, the regional prevalence of COPD symptoms and the utilization of healthcare facilities by COPD patients were studied in 11 countries, 10 of which were from the MENA region [15]. Nevertheless, there is currently a lack of research focusing on the burden of COPD for all countries in the MENA region. Accordingly, the current study aimed to report the burden of COPD and its attributable risk factors in the 21 countries located in the MENA region, by age, sex, and socio-demographic index (SDI), between 1990 and 2019.

## Methods

### Overview

The GBD project is managed by the Institute for Health Metrics and Evaluation (IHME) in Washington (USA) and aims to provide comprehensive and up-to-date information regarding the disease burden and epidemiological patterns across the world. The present study uses GBD 2019 data to report the burden of COPD in the MENA region, and the individual countries within this region, from 1990 to 2019. MENA is comprised of the following 21 countries: Afghanistan, Algeria, Bahrain, Egypt, Iran (Islamic Republic of), Iraq, Jordan, Kuwait, Lebanon, Libya, Morocco, Oman, Palestine, Qatar, Saudi Arabia, Sudan, the Syrian Arab Republic, Tunisia, Turkey, the United Arab Emirates and Yemen. In 2019, the total population of MENA was estimated to be approximately 608.7 million [16]. A detailed description of the methods used to derive the disease burden and to analyse each of the measures in GBD 2019 have been previously published [10, 17]. The results are accessible online using the GBD Results tool (https://ghdx.healthdata.org/gbd-results-tool).

### Case definition

COPD was defined using the GOLD criteria. A diagnosis of COPD is confirmed using spirometry, when the values are < 0.7 FEV1/FVC (the proportion of the maximum amount of air that is forcefully exhaled in the first second of expiration to the total forced expiration volume), following bronchodilation. The severity of COPD was also graded according to the GOLD classification system: mild (I), moderate (II), and severe or very severe combined (III, IV), which are defined as FEV1 scores of ≥ 80%, 50–79%, and < 50% of the normal value, respectively [10]. It is worth nothing that the case definition of COPD was changed to the GOLD criteria from GBD 2013 onwards, with the Lower Limit of Normal (LLN) definition being used in previous iterations of the GBD. LLN categorises COPD according to age- and sex-specific norms for the FEV1/FVC ratio, but since the severity grading of COPD follows the GOLD classification, the reference definition was changed accordingly. The alternative criteria for COPD diagnosis included: GOLD pre-bronchodilation, LLN post-bronchodilation, LLN pre-bronchodilation, and the European Respiratory Society (ERS) guidelines [10].

### Data sources

The data used to estimate COPD mortality was comprised of vital registration and surveillance data from the cause of death database. Verbal autopsy data were not included, since they reported all chronic respiratory diseases combined. Data sources were excluded if they were: (1) implausibly high or low, (2) conflicting substantially with established age or temporal patterns, or (3) conflicting markedly with other data sources obtained from the same locations or locations with similar characteristics [10].

Non-fatal health data required for estimating the burden attributable to COPD were retrieved via a systematic review of published articles, unpublished reports, survey data available in the repository of the Global Health Data Exchange for GBD 2019, and the database of individual-level International Classification of Diseases (ICD)-coded health service encounters from the USA [10]. The detailed search terms used for the systematic review are reported elsewhere [10]. For the GBD 2019 study, no new systematic review of the literature was carried out, but new raw data were added from the English Longitudinal Study of Aging (ELSA) and claims data from the United States. A total of 57 countries, out of the 204 GBD countries and territories, had data available. In terms of data availability for MENA, the non-fatal outcome data covered eight of the countries in this region. Data on the prevalence, incidence, and remission attributable to COPD (defined as the cure rate in GBD), as well as the proportion of each GOLD severity class were extracted when spirometry-based measures were available [10]. The resources included in the disease modeling process are available from this link: http://ghdx.healthdata.org/gbd-2019/data-input-sources.

### Disease model

The Cause of Death Ensemble modeling (CODEm) tool was used to estimate the number of deaths due to COPD for those aged 1–95^+^ years old, for both males and females [10]. The covariates used in the mortality estimation are summarised in a previous publication [10]. For non-fatal health outcomes, after the inclusion of all available data on the prevalence, incidence, remission, as well as cause of death, the burden attributable to COPD was modelled by IHME in two stages: (1) DisMod-MR 2.1 was used to model the prevalence and incidence of COPD, (2) DisMod-MR 2.1 was used to estimate the proportion of each COPD severity using the GOLD class categories. Data from the literature and population representative surveys were split according to age and sex. However, whenever the data were not disaggregated into males and females, or into different age groups, a sex split was implemented using the sex proportions obtained from previous studies with sex specific data and the age categories was divided into smaller age bands using the age pattern of COPD modelled in GBD 2017. Following this, a series of adjustments were made to data that did not use the reference case definition (i.e. GOLD post-bronchodilation criteria) [10]. Hospital claims data were subjected to selection bias, as a result of the wide differences in the socioeconomic status of the countries. Thus, the Burden of Obstructive Lung Disease (BOLD) Study data were used as the reference definition and other claims data were adjusted in accordance with the BOLD findings [10].

The remission data was set to 0, since patients do not recover once they have been affected by COPD, it is only possible to manage the symptoms. Furthermore, in order to avoid an over estimation in age ranges with little or no available data, the incidence ceiling was set at 0.0002 (< 15 years old) and a ceiling at 0.0005 (< 30 years of age). In order to model the prevalence of COPD, a scaler (i.e. standardised exposure variables) was included as a predictive covariate for the combined exposure to all risk factors estimated for COPD, in particular for countries with insufficient data. Furthermore, estimates of the cause-specific mortality rate (CSMR) were included in DisMoD-MR 2.1. In order to derive the estimated excess mortality rate (EMR), every prevalence data point was divided by the CSMR value for that corresponding location, age, sex, and year [10]. Healthcare Access and Quality index (HAQi) and the proportion of elevation over 1500 m altitude were included as country-level covariates for the models, forcing a negative and positive coefficient over the assumption that case fatality decreases with higher wealth levels and increases with higher altitude in a country, respectively. This stage produced the prevalence and incidence of COPD by location, age, sex, and year [10].

In the second stage, data from surveys that stratified the prevalence in accordance with the GOLD severity categories were added to the DisMoD-MR 2.1 model, in order to estimate the overall proportion of COPD cases in each severity level [10].

### Severity splits

DisMod-MR 2.1 was used to model the proportion of people in each GOLD severity class (I, II, III or IV), which were forced to sum to a total of 1 and then multiplied by the overall prevalence of COPD. It is important to note that the GOLD class groupings categorize the COPD severity according to a physiological measurement, rather than according to the clinical presentations of the patients. In order to map prevalence into three COPD health states, based on the GOLD severity classes, IHME used the Medical Expenditure Panel Survey (MEPS) data for 2001–2011 from the USA. MEPS is an ongoing study with data that is updated every 2 years. The individuals with a diagnosis of COPD were identified using the following ICD-10 (J40, J41, J42, J43, J44, and J47) and ICD-9 (490–492, 494 and 496) codes. The scores were then translated from a generic quality-of-life instrument, the 12-Item Short Form Health Survey (SF-12), into the GBD disability weights (DWs). After adjusting for comorbidities, a specific DW was assigned to each individual with a diagnosis of COPD. Cases were categorised into asymptomatic, mild, moderate, and severe COPD, based on the DWs. The different health states were presented as lay descriptions, as shown in Additional file 1: Table S1. In the next step, GOLD class designations, estimated for the USA in 2005 (the midpoint of MEPS years of analyses), were used to map the distribution of cases by GOLD class into the distribution of severity from the MEPS. This led to a mapping from GOLD class into the GBD health states, which could then be applied to the prevalence data by GOLD class from all locations and time periods for both sexes and all age groups [10].

### Compilation of results

The years of life lost (YLLs) were estimated by multiplying the number of deaths in each age group with the remaining life expectancy in that age group, which was sourced from the GBD standard life table. The years lived with disability (YLDs) were produced by multiplying the prevalence of each COPD severity level with their severity-specific DWs. The DALYs were then calculated by summing the YLLs and YLDs. The level of uncertainty was estimated by taking 1000 samples, drawing from the distributions of the sampling error around the data being entered, corrections for measurement error, and estimates of residual non-sampling error. The 25th and 975th values of the ordered draws demarcated the uncertainty intervals (UIs).

Smoothing Splines models were used to investigate the relationship between the burden of COPD (i.e., DALYs) and SDI [18]. SDI ranges from 0 (least developed) to 1 (most developed), and is comprised of the lagged distributed income per capita, average number of years of education over the age of 15 years, and the total fertility rate (< 25 years old). R software (version 3.5.2) was used to present the age-standardised point prevalence, deaths and DALY rates.

### Risk factors

There is robust evidence that COPD can be caused by a number of risk factors [17], including smoking, ambient particulate matter pollution, occupational particulate matter, gases and fumes, household air pollution from solid fuels, exposure to second hand smoke, ambient ozone pollution, low temperatures and high temperatures. The proportion of DALYs attributable to each of the above mentioned risk factors were reported for each country, as well as by sex and age group. A description of these risk factors, and their relative risks, are available elsewhere [17].

## Results

### The Middle East and North Africa region

In 2019, there were an estimated 10.7 million (95% UI: 10.3, 11.2) cases of COPD, with an age-standardised point prevalence of 2333.9 per 100,000 (95% UI: 2230.1, 2443.6), which was 30.6% (95% UI: 28.2%, 33.0%) higher than in 1990 (Table 1 and Additional file 2: Table S2). COPD accounted for more than 89.9 thousand deaths (95% UI: 76.8, 101.8) with an age-standardised death rate of 26.1 (95% UI: 22.2, 29.5) per 100,000, which was 18.0% (95% UI: 2.8%, 30.9%) lower than in 1990 (Table 1 and Additional file 3: Table S3). Furthermore, in 2019 the COPD-related DALYs were 2.8 million (95% UI: 2.4, 3.1), with an age-standardised rate of 649.1 (95% UI: 574.6, 717.7) per 100,000, which decreased by 11.8% (95% UI: 0.9%, 21.1%) between 1990 and 2019 (Table 1 and Additional file 4: Table S4).

**Table 1:** Prevalent cases, deaths and DALYs due to chronic obstructive pulmonary disease in the Middle East and North Africa region in 2019 and the percentage change in the age-standardised rates during the period 1990–2019.

	Prevalence (95% UI)			Deaths (95% UI)			DALYs (95% UI)		
	Counts (2019)	ASRs (2019)	Pcs in ASRs 1990–2019	Counts (2019)	ASRs (2019)	Pcs in ASRs 1990–2019	Counts (2019)	ASRs (2019)	Pcs in ASRs 1990–2019
North Africa and Middle East	10,720,230 (10,262,918, 11,204,177)	2333.9 (2230.1, 2443.6)	30.6 (28.2, 33)	89,920 (76,785, 101,801)	26.1 (22.2, 29.5)	− 18 (− 30.9, − 2.8)	2,761,836 (2,436,839, 3,062,623)	649.1 (574.6, 717.7)	− 11.8 (− 21.1, − 0.9)
Afghanistan	371,175 (349,917, 393,665)	2141.4 (1998, 2291.4)	21.4 (16.8, 25.9)	3932 (2506, 5144)	40.4 (24.2, 52.6)	− 15.7 (− 36.2, 7.8)	137,887 (100,493, 173,594)	964.5 (681.8, 1203.2)	− 14.1 (− 32.8, 7.4)
Algeria	628,753 (590,277, 667,208)	1787.8 (1682, 1899.8)	24 (19.8, 28.1)	4905 (3836, 6207)	20.5 (16, 25.7)	− 29.9 (− 45.6, − 8.7)	148,252 (124,597, 175,872)	465.1 (390.8, 548)	− 19.7 (− 34, − 1.9)
Bahrain	20,177 (18,350, 22,134)	2013.4 (1880.4, 2151.1)	− 22.1 (− 25.2, − 18.8)	111 (88, 142)	28.9 (23.5, 35.7)	− 52.3 (− 62.3, − 37.4)	4014 (3426, 4797)	561.7 (484.6, 662.1)	− 51.1 (− 59, − 39.5)
Egypt	1,881,259 (1,787,452, 1,965,262)	2731 (2588.1, 2872)	62 (55.6, 68.9)	14,052 (9238, 18,627)	28.2 (18.5, 36.8)	− 2.6 (− 27.6, 30.7)	499,452 (378,041, 615,259)	776.4 (583.1, 954.9)	8.2 (− 12.2, 34.3)
Iran (Islamic Republic of)	1,562,415 (1,446,402, 1,688,536)	2055.5 (1891.4, 2232.6)	25.3 (20.9, 29.8)	12,557 (10,993, 13,621)	20.3 (17.7, 22.1)	− 13 (− 30.5, 1.7)	372,569 (338,458, 404,911)	517.2 (471, 560.8)	− 7 (− 20.6, 3.2)
Iraq	308,960 (281,559, 338,889)	1053.5 (976.7, 1139.4)	− 28.9 (− 32.7, − 24.4)	1711 (1343, 2143)	9.8 (7.8, 12.6)	− 15.9 (− 41, 12.6)	65,299 (54,269, 76,574)	265.2 (221.3, 311.2)	− 23.2 (− 40.1, − 7)
Jordan	119,919 (110,359, 129,523)	1602.3 (1504.3, 1706.6)	− 8.1 (− 12.3, − 3.6)	555 (450, 680)	12.2 (9.9, 15)	− 53 (− 64.7, − 37.4)	22,410 (19,437, 25,914)	341.2 (297.1, 392.9)	− 42.9 (− 53.4, − 30.7)
Kuwait	39,087 (34,844, 43,652)	1023.1 (939.3, 1115.6)	2.8 (− 1.4, 6.9)	148 (117, 182)	8.3 (6.5, 10.3)	− 12.7 (− 30.8, 8.4)	6186 (5236, 7195)	215.7 (185.7, 249.7)	− 12 (− 23.7, 1.9)
Lebanon	141,237 (131,990, 150,458)	2723.1 (2548.3, 2901.7)	43 (38.1, 47.8)	850 (631, 1107)	16.8 (12.6, 21.8)	− 21.8 (− 40.2, 2)	27,283 (22,613, 31,817)	525.6 (436.8, 612.6)	− 3 (− 18.7, 13.8)
Libya	139,857 (131,289, 147,932)	2461 (2306.8, 2612.1)	43.6 (38.1, 49.8)	760 (561, 973)	17.6 (13.1, 22.5)	− 1.2 (− 30.3, 34.3)	28,042 (23,234, 33,105)	531.5 (440.8, 630.6)	13.7 (− 9.4, 38.6)
Morocco	611,177 (573,610, 650,326)	1896.7 (1782.4, 2018.8)	38.9 (33.4, 44.9)	5935 (4494, 7387)	24.1 (18.6, 29.7)	11.1 (− 15.1, 49.9)	172,958 (141,768, 203,952)	577.5 (476.2, 680.6)	13.4 (− 7, 39)
Oman	33,346 (28,850, 38,642)	1281.2 (1172.5, 1395.8)	46.2 (39.6, 53.6)	202 (155, 235)	23 (16.8, 27.2)	− 32.7 (− 52.1, − 9.3)	7193 (6122, 8214)	453.9 (363.5, 514.4)	− 32.6 (− 50.4, − 12.1)
Palestine	44,568 (40,888, 48,163)	1552.7 (1454.1, 1659.8)	− 9.1 (− 12.9, − 5.2)	285 (234, 368)	16.4 (13.4, 21.4)	− 36.5 (− 61.4, − 14)	9932 (8572, 11,625)	414.3 (359.2, 492.5)	− 31.5 (− 52.4, − 14)
Qatar	26,415 (23,313, 29,791)	1754.9 (1633.4, 1886.3)	− 11.4 (− 15.2, − 7.7)	49 (36, 77)	20.2 (15.5, 28.6)	− 24.1 (− 47.2, 1.8)	3730 (3055, 4557)	410.9 (345.6, 522.7)	− 30.8 (− 45.7, − 15.2)
Saudi Arabia	434,561 (396,012, 473,597)	2053 (1918.1, 2194.3)	48.6 (42.5, 54.8)	2119 (1685, 2576)	19.6 (15.9, 23.4)	− 41.4 (− 61.9, − 20)	91,982 (77,925, 107,172)	508.2 (434.9, 581.6)	− 25.3 (− 47.4, − 4.4)
Sudan	428,128 (401,529, 455,381)	1862.6 (1748, 1989.1)	18.3 (14.1, 23.6)	4390 (2883, 6190)	28.7 (18.9, 40.1)	− 21.5 (− 41.2, 6.9)	137,492 (102,426, 179,883)	682.3 (500.3, 897.6)	− 19.7 (− 36.9, 4)
Syrian Arab Republic	249,284 (233,862, 265,450)	2017.3 (1903, 2137.8)	22.2 (18.3, 26.1)	1908 (1367, 2781)	20.9 (15.4, 30.4)	− 4.8 (− 30.6, 33.8)	64,521 (52,675, 80,806)	546.8 (448, 686.7)	− 2.8 (− 23.5, 25.1)
Tunisia	249,513 (233,445, 266,354)	2048.5 (1916.8, 2186.2)	32.7 (27.7, 38.2)	1831 (1325, 2497)	16.8 (12.1, 22.9)	− 12 (− 35.3, 18.2)	54,631 (44,085, 67,863)	454.7 (367.8, 563)	0.3 (− 19.1, 23.8)
Turkey	2,885,498 (2,798,099, 2,967,100)	3287.1 (3187.4, 3380.3)	28.8 (24.4, 33.2)	29,015 (19,528, 35,902)	35.8 (24, 44.4)	− 26.1 (− 46.4, − 4.1)	733,647 (575,036, 858,889)	855.2 (667.5, 1000.4)	− 22.2 (− 36.7, − 6.1)
United Arab Emirates	191,068 (187,010, 194,836)	2926.9 (2811.1, 3046.4)	29.9 (24.4, 36.4)	1090 (653, 1561)	31.2 (22.8, 41.3)	− 25.6 (− 50.4, 5.3)	59,161 (39,649, 78,876)	886.1 (698.8, 1101.3)	− 11.5 (− 35.9, 19.3)
Yemen	342,941 (323,483, 363,074)	2065 (1932.8, 2207)	19.2 (13.9, 24)	3423 (2591, 4521)	33 (25, 42.9)	− 17.8 (− 41.7, 16)	112,389 (90,488, 140,804)	785.1 (634.1, 976)	− 14.6 (− 36.3, 14.5)

Generated from data available from http://ghdx.healthdata.org/gbd-results-tool

### National level

**Fig. 1:**
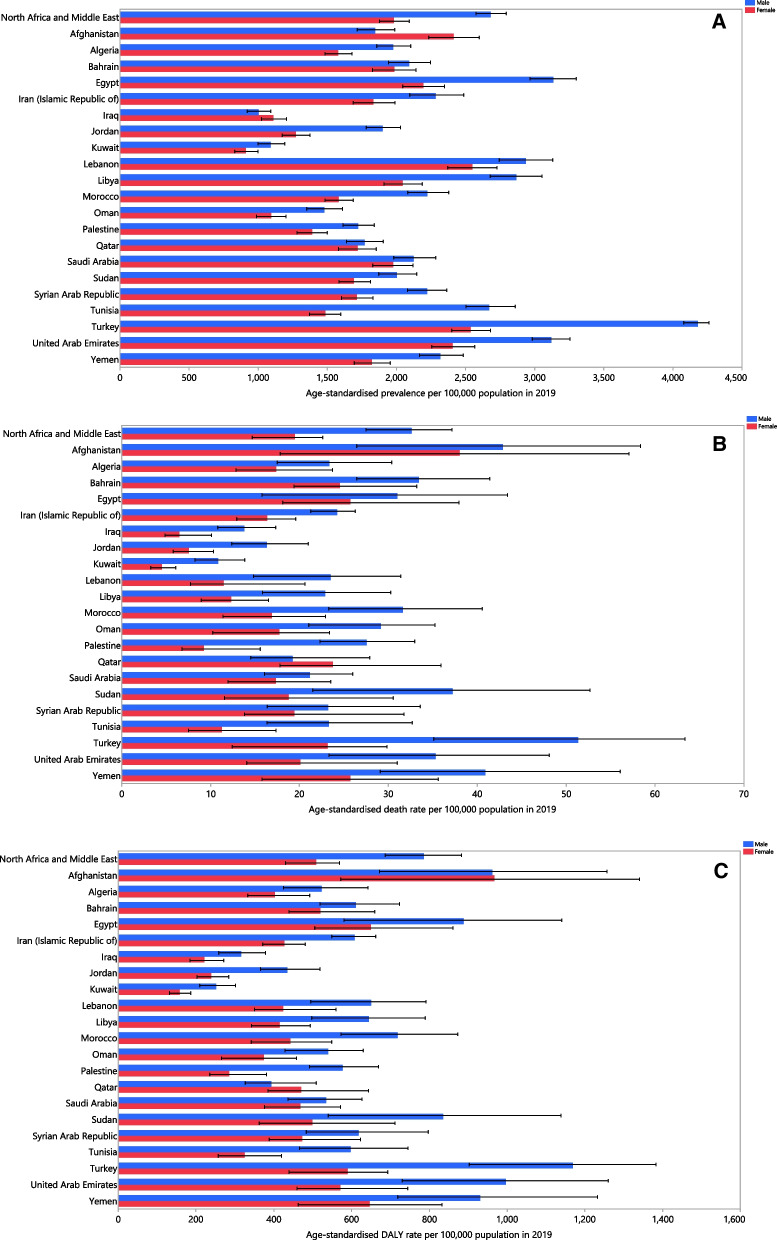
Age-standardised point prevalence (**A**), death (**B**), and DALYs (**C**) of chronic obstructive pulmonary disease (per 100,000 population) in the Middle East and North Africa region in 2019, by sex and country. *DALY* disability-adjusted-life-years (Generated from data available from http://ghdx.healthdata.org/gbd-results-tool)

The age-standardised point prevalence of COPD varied significantly between countries in the MENA region. Turkey [3287.1 (95% UI: 3187.4, 3380.3)], the UAE [2926.9 (95% UI: 2811.1, 3046.4)] and Egypt [2731.0 (95% UI: 2588.1, 2872.0)] had the highest age-standardised point prevalence per 100,000 population. In contrast, Kuwait [1023.1 (95% UI: 939.3, 1115.6)], Iraq [1053.5 (95% UI: 976.7, 1139.4)] and Oman [1281.2 (95% UI: 1172.5, 1395.8)] had the lowest rates (Fig. 1A and Additional file 2: Table S2). Afghanistan [40.4 (95% UI: 24.2, 52.6)], Turkey [35.8 (95% UI: 24.0, 44.4)] and Yemen [33.0 (95% UI: 25.0, 42.9)] had the three highest age-standardised death rates in 2019, whereas Kuwait [8.3 (95% UI: 6.5, 10.3)], Iraq [9.8 (95% UI: 7.8, 12.6)] and Jordan [12.2 (95% UI: 9.9, 15.0)] had the lowest (Fig. 1B and Additional file 3: Table S3). The highest age-standardised DALY rates were observed in Afghanistan [964.5 (95% UI: 681.8, 1203.2)], the UAE [886.1 (95% UI: 698.8, 1101.3)] and Turkey [855.2 (95% UI: 667.5, 1000.4)]. In contrast, Kuwait [215.7 (95% UI: 185.7, 249.7)], Iraq [265.2 (95% UI: 221.3, 311.2)] and Jordan [341.2 (95% UI: 297.1, 392.9)] had the lowest age-standardised DALY rates in the MENA region (Fig. 1C and Additional file 4: Table S4).

There were substantial inter-country differences in the percentage change in the estimated age-standardised point prevalence from 1990 to 2019. Egypt [62.0% (95% UI: 55.6, 68.9)], Saudi Arabia [48.6% (95% UI: 42.5, 54.8)] and Oman [46.2% (95% UI: 39.6, 53.6)] had the highest increases. In contrast, Iraq [− 28.9% (95% UI: − 32.7, − 24.4)], Bahrain [− 22.1% (95% UI: − 25.2, − 18.8)] and Qatar [− 11.4% (95% UI: − 15.2, − 7.7)] had the largest decreases in the age-standardised point prevalence (Additional file 2: Table S2 and Additional file 5: Fig. S1). For the change in the age-standardised death rate, Jordan [− 53.0% (95% UI: − 64.7, − 37.4)], Bahrain [− 52.3% (95% UI: − 62.3, − 37.4)] and Saudi Arabia [− 41.4% (95% UI: − 61.9, − 20.0)] had the largest decrease over the period 1990–2019 (Additional file 3: Table S3 and Additional file 6: Fig. S2). Similarly, Bahrain [− 51.1% (95% UI: − 59.0, − 39.5)], Jordan [− 42.9% (95% UI: − 53.4, − 30.7)] and Oman [− 32.6% (95% UI: − 50.4, − 12.1)] demonstrated the largest decreases in the age-standardised DALY rates during the measurement period (Additional file 4: Table S4 and Additional file 7: Fig. S3).

### Age and sex patterns

**Fig. 2:**
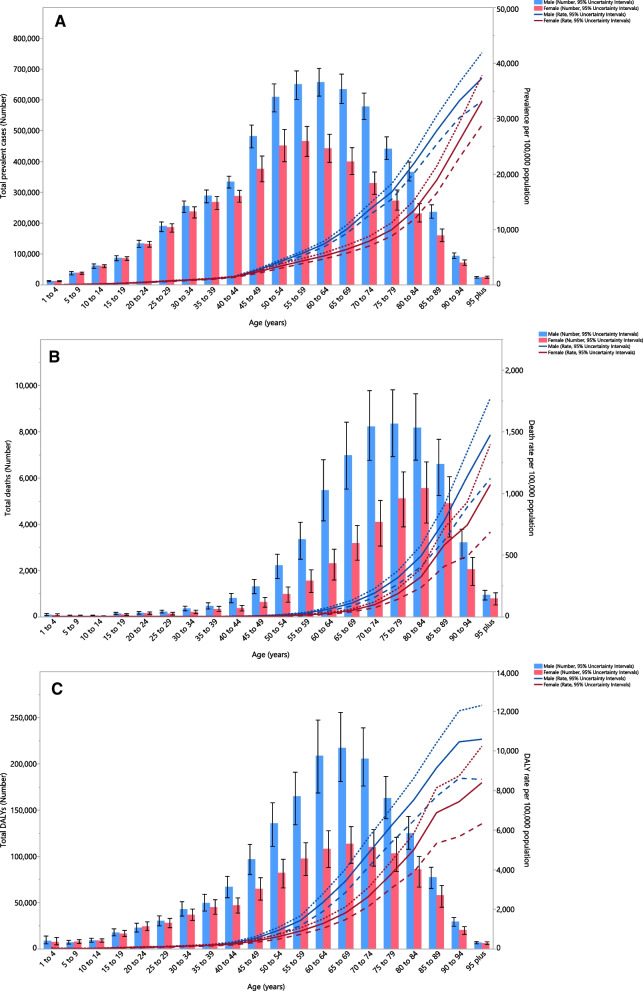
Numbers of prevalent cases and prevalence (**A**), number of deaths and death rate (**B**) and the number of DALYs and DALY rate (**C**) for chronic obstructive pulmonary disease per 100,000 population in the Middle East and North Africa region, by age and sex in 2019; dotted and dashed lines indicate 95% upper and lower uncertainty intervals, respectively. *DALY* disability-adjusted-life-years (Generated from data available from http://ghdx.healthdata.org/gbd-results-tool)

In 2019, the total number of prevalent cases in males increased with population aging, from the lowest prevalence in those aged 1–4 age years to a peak in the 60–64 age group, before decreasing again with advancing age. A similar pattern was observed for females, but the total number of prevalent cases reached its highest in the 55–59 age group and then decreased with increasing age. Furthermore, the prevalence of COPD was lower in females in all age groups, except for those older than 95 years old. In contrast, the point prevalence per 100,000 increased with age for both sexes. The point prevalence was similar for both sexes, up to 45 years of age, but in older adults it was again higher among males (Fig. 2A).

The total number of deaths due to COPD, in both sex groups, increased slowly with age with a steep increase in people older than 45 years, followed by a slight decrease. The highest number of deaths in males and females were in those aged 75–79 and 80–84 years, respectively, with the lowest number of deaths being in the 5–9 and 10–14 age groups, for both sexes. However, the death rate increased constantly with age, for both sexes, with a higher rate in males in all age groups (Fig. 2B). The total number of DALYs associated with COPD increased with age, peaking in the 65–69 age groups, followed by decreases in the older age groups, in both sexes. Except for 5–9 and 20–24 years old, males had higher DALYs than females. The DALY rate also consistently increased with aging, in both sex groups, with a higher slope in people older than 49 years of age (Fig. 2C).

**Fig. 3:**
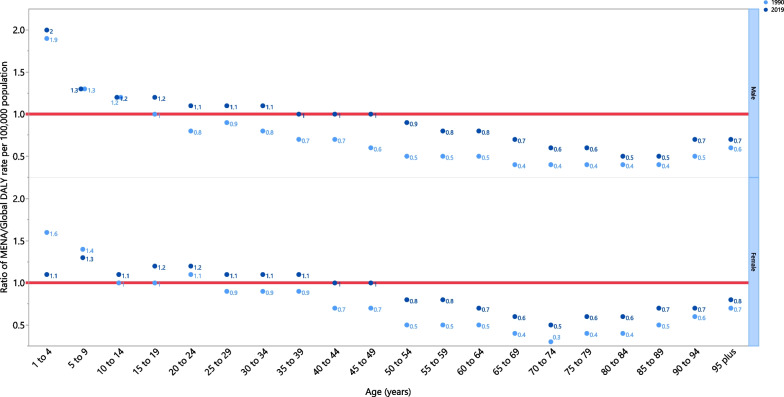
Ratio of the Middle East and North Africa region to the global chronic obstructive pulmonary disease DALY rate according to age group and sex, 1990–2019. *DALY* disability-adjusted-life-years (Generated from data available from http://ghdx.healthdata.org/gbd-results-tool)

In 2019, the COPD DALY rate was lower than the global DALY rate (ratio of MENA/global DALY rate < 1) in people older than 50 years, for both males and females. In females, people aged 40–49 had DALY rates similar to the global rate (ratio of MENA/global DALY rate = 1), while females younger than 39 had a higher DALY rate. Males had a DALY rate equal to the global rate in the 35–49 age group, but males younger than 34 had higher DALY rates. Furthermore, the DALY rate in males aged 1–4 was twice the global average. In 2019, males had higher DALY rates in all age groups, except for males aged 5–14, which was the same as the global average. Also in 2019, the DALY rate for females was lower than the global average for the 1–4 and 5–9 age groups, but was higher in all other age groups (Fig. 3).

### Association with socio-demographic index (SDI)

**Fig. 4:**
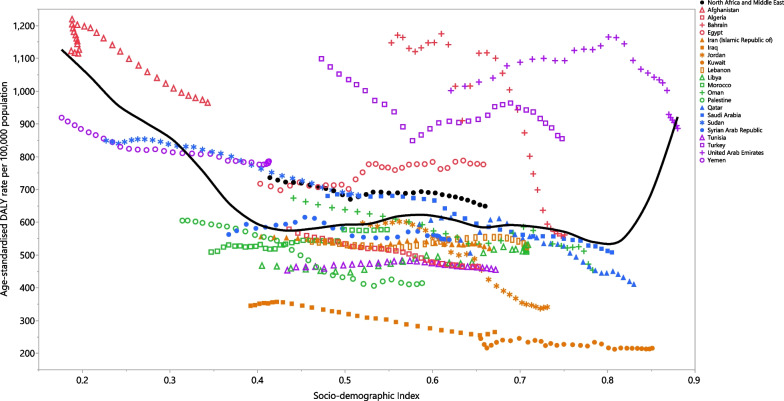
Age-standardised DALY rates of chronic obstructive pulmonary disease for 21 countries and territories in the Middle East and North Africa region, by SDI in 2019; Expected values based on the Socio-demographic Index and disease rates in all locations are shown as the black line. Each point shows the observed age-standardised DALY rate for each country in 2019. *DALY* disability-adjusted-life-years, *SDI* Socio-demographic Index (Generated from data available from http://ghdx.healthdata.org/gbd-results-tool)

At the regional level, a non-linear U-shaped association was observed between SDI and the DALY rate of COPD from 1990 to 2019 (Fig. 4). Generally, the MENA countries experienced a decrease in the DALY rate with increasing SDI levels. Afghanistan, Egypt and Turkey had higher than expected rates from 1990 to 2019, while Kuwait, Iraq, Libya, Palestine, Jordan, Tunisia, Iran, Algeria, Lebanon and Morocco had lower than expected rates, based upon their SDI level. Oman, Saudi Arabia, Bahrain and the UAE reached a lower than expected rate during the measurement period, whereas Yemen and Sudan reached a higher than expected rate during the measurement period, despite having a decreasing trend. After slight increases, and reaching higher than expected rates, Qatar and Syria had lower than expected rates with a decreasing trend (Fig. 4).

### Risk factors

**Fig. 5:**
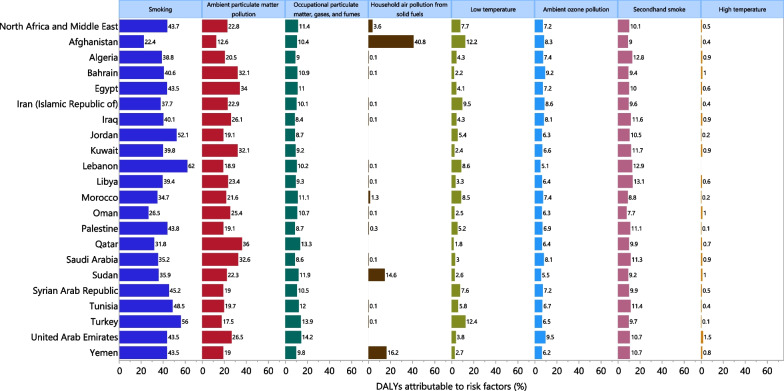
Percentage of DALYs due to chronic obstructive pulmonary disease attributable to risk factors for the Middle East and North Africa countries in 2019. *DALY* disability-adjusted-life-years (Generated from data available from http://ghdx.healthdata.org/gbd-results-tool)

Despite large national and sex differences, smoking (43.7%), ambient particulate matter pollution (22.8%) and occupational particulate matter (11.4%) had the largest proportion of the attributable DALYs for COPD in 2019 (Fig. 5). Smoking (62.0%), ambient particulate matter pollution (23.3%) and occupational particulate matter (16.1%) had the highest attributable burden in males, whereas ambient particulate matter pollution (22.1%), smoking (15.1%) and second-hand smoking (12.5%) were the main risk factors for COPD in females (Additional file 8: Fig. S4).

## Discussion

This study was conducted to estimate the burden of COPD and its attributable risk factors in the MENA region. Globally, the GBD 2019 findings showed that COPD was the 6th leading cause of DALY in all age groups, the 4th leading cause of mortality in people aged 50–74 and the 3rd in people older than 75 years of age [10]. Regionally, the age-standardised point prevalence of COPD increased by 30.6%, while the age-standardised rates for both the death and DALY decreased by 18% and 11.8%, respectively, between 1990 and 2019. Generally, the burden of COPD was positively associated with age and had a non-linear U-shaped association with SDI level.

A previous study reported global decreases in the age-standardised point prevalence, death and DALY rates per 100,000 for all chronic respiratory diseases (including asthma, COPD etc.) between 1990 and 2017, which is consistent with our result for the death and DALY rates, but not for the point prevalence. This slight discrepancy may be due to different trends in the GBD super-regions, or may be as a result of reporting the findings for all chronic respiratory diseases combined [9].

In comparison with other countries, in 2019 Turkey had the first, second and third highest rank in the age-standardised point prevalence, death and DALY rates attributable to COPD, respectively. This could be as a result of historically high tobacco smoking in Turkey. GBD 2019 data estimated that Turkey had one of the highest age-standardised point prevalence rates of tobacco smoking, especially in females, which was nearly three-times the global prevalence [19]. Furthermore, the current study showed that the second largest proportion of DALYs attributable to smoking was found in Turkey, with 56%. Also, the proportion of DALYs attributed to tobacco smoking in Turkish males and females were the highest (71.2%) and second highest (30.0%) in MENA, respectively. The highest mortality rates were observed in Afghanistan, Turkey and Yemen. In addition to the high prevalence of COPD in Turkey, which explains the high mortality rate, the high mortality rates in Afghanistan and Yemen, despite relatively low prevalence, are likely to be due to poor access to both preventive and treatment healthcare services, partially as a result of the armed conflicts in these two countries [20, 21]. The reconstruction of the health system, during and after the conflicts, and providing a safer context for community healthcare providers and a safer supply chain should be the main objectives in these countries [22, 23]. In comparison with other countries in this region, Kuwait had the lowest age-standardised mortality and DALY rates. The low mortality and DALY rates in Kuwait are due to the low proportion of elderly people (i.e. older than 65), significant economic prosperity and the substantial investment in the healthcare system. As a result, Kuwait has one of the best healthcare systems in the Persian Gulf [24]. Large differences in the cultural, sociodemographic and economic status of the countries in MENA may also influence the burden of COPD. For example, citizens in less developed, and war-torn countries such as Afghanistan, where most people live in rural areas with poor healthcare access, are likely to have different lifestyles and health-related behaviors than citizens of more developed countries like Kuwait, where most people live in industrialised cities and have better access to health services. These differences are likely to account for the large heterogeneity in the prevalence, mortality and burden of this disease among the MENA countries.

Previous research has found that COPD mortality rates increase with increasing age, which is in accordance with our results [25]. Moreover, our results showed that the DALY rates due to COPD in people older than 50 years of age, who had the highest prevalent cases, were lower than the global rates. This could be as a result of the lower age-standardised prevalence of tobacco smoking in MENA (than globally), since smoking is the main risk factor for developing COPD and/or a better than universal health coverage index in MENA [19, 26].The DALY rates among young children in MENA were higher than the global average, but to our knowledge no previous research has investigated COPD among children in this region.

This study observed an almost U-shaped association between the DALY rates and the sociodemographic index (SDI), which is in agreement with previous studies [12, 27]. However, a positive association was observed between SDI and the DALY rates at SDI levels higher than 0.8 and since UAE is the only country with an SDI higher than 0.8, there was a higher than expected DALY rate in this country. Furthermore, this positive association can be attributed to the high DALY rates in this country, which may be as a result of the high number of expatriate workers, and thus a high male/female ratio, as well as a high growth rate in the number of elderly people [28], although the DALY rate in this country started to decrease with increasing SDI over the last few years. Moreover, the higher contribution of occupational risks, smoking and high body mass index in countries with low SDI levels could lead to an increase in the age-standardised death rates of COPD in these countries [12].

Our study showed that tobacco smoking, ambient particulate matter pollution and occupational particulate matter were the main risk factors for developing COPD in the MENA region. Smoking was the largest risk factor in males, whereas ambient particulate matter pollution ranked first among females. In the 2013 Global Tobacco Control Report by the World Health Organisation (WHO), Turkey was reported to be the first country to achieve a high level of success in the six MPOWER strategies, and other countries were advised to adopt the Turkish policies. These six strategies include: monitor tobacco use, protect people from tobacco smoke, offer help to stop tobacco use, warn about the dangers of tobacco, enforce bans on tobacco advertising and promotion, raise taxes on tobacco products [29]. According to the WHO report on the global tobacco epidemic 2021, the Turkish government had implemented some policies to limit tobacco use, such as plain packaging of tobacco products, national mass media efforts and putting all MPOWER measures in place at a comprehensive level [30]. GOLD 2022 reports pulmonary rehabilitation to be the most effective therapeutic strategy to improve the signs and symptoms of COPD and notes the establishment of 35 rehabilitation centers in Turkey in 2017. In combination with the above, and the therapeutic and political measures, could result in a future reduction of the burden of COPD in Turkey [31]. Adopting similar policies in other countries in the region with a high prevalence of tobacco smoking would also decrease the incidence of COPD, and result in a subsequent decrease in the burden of this disease.

Second to tobacco smoking, ambient particulate matter pollution is a common cause of COPD among both sexes in the MENA region. Ambient particulate matter is also the most common risk factor for developing COPD in Qatar, in which extreme weather conditions (e.g. sand storms) and high anthropogenic emissions play a significant role in increasing the ambient particulate matter levels [32]. Egypt is the country with the largest increase in the age-standardised point prevalence and the third largest increase in the DALYs due to COPD between 1990 and 2019, and Egypt’s capital, Cairo, is one of the most polluted metropolitan areas in the world. Therefore, air pollution due to increasing industrialisation and massive fossil fuel combustion plays a great substantial role in the occurrence of this increase [33]. Saudi Arabia is the country with the second highest increase in the age-standardised point prevalence in MENA. Similar to Egypt Saudi Arabia has several industrialised cities causing substantial air pollution which has likely led to the increase prevalence of COPD over two decades, although the age-standardised death rate in Saudi Arabia decreased markedly over this period, which is probably due to an increase in the quality of healthcare provision [34]. Clearly the implementation of national policies to decrease the production of particulate matter and encouraging people to avoid polluted air, by staying at home or using masks, could benefit these countries [35].

Household air pollution from solid fuels is the main risk factor for developing COPD in females and a major risk factor in low-income countries within MENA. This is particularly a problem in Afghanistan, Yemen and Sudan. In contrast with Turkey, and most other countries in MENA, tobacco smoking makes little contribution to the development of COPD in Afghanistan, while household air pollution is the main risk factor. The association between household air pollution from burning solid fuels (e.g., wood, animal dung, agricultural crop residues and biomass fuel) and COPD has been previously established [36]. According to data from the GBD 2016, COPD was the 4th largest cause of death and DALYs in Afghan males older than 70 years of age [37]. Household air pollution was also the second largest attributable risk factor for all causes of DALYs among Afghan people, demonstrating the high usage of solid fuels and explaining the rationale for household air pollution being the main risk factor for COPD among Afghan people [37]. Previous studies have shown the benefits of taking a preventive approach, such as using clean fuels or electricity for cooking or warming, but this may not be applicable in many low-income countries. Therefore outdoor cooking with biomass fuels, partitioning the kitchen from other parts of the house or using better stoves with more efficient chimneys could reduce the burden of this risk factor [38, 39].

Occupational exposures were the third most common risk factor for the burden attributable to COPD in MENA. Iraq (8.4%) and the UAE (14.2%) had the lowest and highest burden of COPD attributable to occupational exposures in MENA, respectively. In addition, the burden of COPD attributable to occupational exposures were almost four times higher in males than females in the MENA region (16.1% vs. 4.1%). In line with our findings, the global burden of COPD attributable to occupational exposures were reported to be approximately two times higher in males than among females (19.2% vs. 10.9%) [17]. The sex differences in the burden attributable to COPD can be explained by the fact that males are at higher exposure to hazards than females [40]. Although the burden of COPD attributable to occupational particles decreased in all regions, it is still a major contributor to the COPD burden globally [17]. Therefore, developing preventive strategies by risk evaluation, exposure reduction and educational programs for workers, as well as early diagnosis and management of COPD, are recommended to reduce the burden of COPD [41]. These strategies are especially important in countries with high attributable burdens to occupational exposures, like the UAE, Turkey and Qatar.

To our best of knowledge, our study is the first study to report the burden of COPD and its attributable risk factors in MENA and to provide the estimates by age, sex and level of socioeconomic development. However, as with all research, this study had a number of limitations. Firstly, scarcity of high-quality primary data, especially in the lower-income countries of the region, could reduce the accuracy and reliability of the available data and thus estimations. Improving healthcare data registry systems can strengthen the precision of such estimations. Furthermore, data has not been reported at the subnational level, so future research is needed to report data at the sub-national level, particularly in relation to the area of residence (i.e., rural or urban) could help policymakers to apply more specific and effective measures. Secondly, the analyses reported in this study were dependent on the data supplied by the GBD project, so we were not able to investigate other important spirometry-based questions, like the proportion of the population who have restrictive impairment that meets the new COPDGene definition of COPD. Thirdly, it seems that some countries like Iraq, the UAE, Bahrain and Turkey had extremely high or low attributable age-standardised DALY rates of COPD, which can substantially affect the overall relationship with SDI. This should be considered when interpreting the results.

## Conclusions

COPD is one of the leading causes of death and disability in the MENA region, but there have been decreases in the age-standardised death and DALY rates over the last three decades. Nevertheless, COPD accounts for a large number of deaths and DALYs, especially among the elderly. Smoking and ambient particular matter pollution were the most common risk factors. Therefore, policymakers and the health systems should aim to reduce these risk factors in order to decrease the incidence of new COPD cases, alongside providing more accurate diagnosis and better care for extant COPD patients. Further studies are needed to estimate the prevalence and burden of this disease more accurately.

## Supplementary Information

**Additional file 1: Table S1.** Health states for chronic obstructive pulmonary disease and the associated disability weights from the Global Burden of Disease 2019 Study.

**Additional file 2: Table S2.** Prevalence of chronic obstructive pulmonary disease in 1990 and 2019 for both sexes and percentage change in age-standardised rates (ASRs) per 100,000 in the Middle East and North Africa region (generated from data available from http://ghdx.healthdata.org/gbd-results-tool).

**Additional file 3: Table S3.** Deaths of chronic obstructive pulmonary disease in 1990 and 2019 for both sexes and percentage change in age-standardised rates (ASRs) per 100,000 in the Middle East and North Africa region (generated from data available from http://ghdx.healthdata.org/gbd-results-tool).

**Additional file 4: Table S4.** DALYs due to chronic obstructive pulmonary disease in 1990 and 2019 for both sexes and percentage change in age-standardised rates (ASRs) per 100,000 in the Middle East and North Africa region (generated from data available from http://ghdx.healthdata.org/gbd-results-tool).

**Additional file 5: Figure S1.** The percentage change in the age-standardised point prevalence of chronic obstructive pulmonary disease in the Middle East and North Africa region from 1990 to 2019, by sex and country (generated from data available from http://ghdx.healthdata.org/gbd-results-tool).

**Additional file 6: Figure S2.** The percentage change in the age-standardised death of chronic obstructive pulmonary disease in the Middle East and North Africa region from 1990 to 2019, by sex and country (generated from data available from http://ghdx.healthdata.org/gbd-results-tool).

**Additional file 7: Figure S3.** The percentage change in the age-standardised DALYs of chronic obstructive pulmonary disease in the Middle East and North Africa region from 1990 to 2019, by sex and country. DALY = disability-adjusted-life-years (generated from data available from http://ghdx.healthdata.org/gbd-results-tool).

**Additional file 8: Figure S4.** Percentage of DALYs due to chronic obstructive pulmonary disease attributable to risk factors for the Middle East and North Africa countries, by sex, in 2019. DALY = disability-adjusted-life-years (generated from data available from http://ghdx.healthdata.org/gbd-results-tool).

## Data Availability

The data used for these analyses are all publicly available at http://ghdx.healthdata.org/gbd-results-tool.
