# Supplementary material for: The burden of chronic obstructive pulmonary disease and its attributable risk factors in the Middle East and North Africa region, 1990–2019
Source: Respir Res. 2022 Nov 19;23:319. doi: 10.1186/s12931-022-02242-z (PMC9675283; doi:10.1186/s12931-022-02242-z)
Supplement: Supplementary file 1 — Additional file 1: Table S1. Health states for chronic obstructive pulmonary disease and the associated disability weights from the Global Burden of Disease 2019 Study. [file 12931_2022_2242_MOESM1_ESM.docx]

| **Table S1: Health states for chronic obstructive pulmonary disease and the associated disability weights from the Global Burden of Disease 2019 Study** | | |
| --- | --- | --- |
| **Health state** | **Lay description** | **Disability weight**  **(95% CI)** |
| Mild COPD | This person has a cough and shortness of breath after heavy physical activity, but is able to walk long distances and climb stairs. | 0.019  (0.011–0.033) |
| Moderate COPD | This person has a cough, wheezing, and shortness of breath, even after light physical activity. The person feels tired and can walk only short distances or climb only a few stairs. | 0.225  (0.153–0.31) |
| Severe COPD | This person has a cough, wheezing, and shortness of breath all the time. The person has great difficulty walking even short distances or climbing any stairs, feels tired when at rest, and is anxious. | 0.408  (0.273–0.556) |
