# Supplementary material for: The burden of chronic obstructive pulmonary disease and its attributable risk factors in the Middle East and North Africa region, 1990–2019
Source: Respir Res. 2022 Nov 19;23:319. doi: 10.1186/s12931-022-02242-z (PMC9675283; doi:10.1186/s12931-022-02242-z)
Supplement: Supplementary file 2 — Additional file 2: Table S2. Prevalence of chronic obstructive pulmonary disease in 1990 and 2019 for both sexes and percentage change in age-standardised rates (ASRs) per 100,000 in the Middle East and North Africa region (generated from data available from http://ghdx.healthdata.org/gbd-results-tool). [file 12931_2022_2242_MOESM2_ESM.docx]

| **Table S2: Prevalence of chronic obstructive pulmonary disease in 1990 and 2019 and the percentage change in the age-standardised rates (ASRs) per 100,000 in the Middle East North and Africa region**  **(Generated from data available from http://ghdx.healthdata.org/gbd-results-tool)** | | | | | |
| --- | --- | --- | --- | --- | --- |
|  | **1990** | | **2019** | | **Percentage change in ASRs per 100,000** |
|  | **No (95% UI)** | **ASRs per 100,000 (95% UI)** | **No (95% UI)** | **ASRs per 100,000 (95% UI)** |  |
| **North Africa and Middle East** | **3584089 (3404769 , 3768097)** | **1787.6 (1690.9 , 1887.5)** | **10720230 (10262918 , 11204177)** | **2333.9 (2230.1 , 2443.6)** | **30.6 (28.2 , 33)** |
| **Afghanistan** | **134721 (124810 , 145783)** | **1764 (1635.3 , 1913.3)** | **371175 (349917 , 393665)** | **2141.4 (1998 , 2291.4)** | **21.4 (16.8 , 25.9)** |
| **Algeria** | **210235 (196977 , 222964)** | **1442.2 (1355.9 , 1534.2)** | **628753 (590277 , 667208)** | **1787.8 (1682 , 1899.8)** | **24 (19.8 , 28.1)** |
| **Bahrain** | **5335 (4908 , 5750)** | **2584.5 (2430.2 , 2743.5)** | **20177 (18350 , 22134)** | **2013.4 (1880.4 , 2151.1)** | **-22.1 (-25.2 , -18.8)** |
| **Egypt** | **588953 (556275 , 622076)** | **1685.4 (1581.6 , 1790.5)** | **1881259 (1787452 , 1965262)** | **2731 (2588.1 , 2872)** | **62 (55.6 , 68.9)** |
| **Iran (Islamic Republic of)** | **509008 (465525 , 555495)** | **1640 (1508.2 , 1794.9)** | **1562415 (1446402 , 1688536)** | **2055.5 (1891.4 , 2232.6)** | **25.3 (20.9 , 29.8)** |
| **Iraq** | **144140 (135387 , 152870)** | **1481.2 (1390.1 , 1578)** | **308960 (281559 , 338889)** | **1053.5 (976.7 , 1139.4)** | **-28.9 (-32.7 , -24.4)** |
| **Jordan** | **31615 (29675 , 33533)** | **1744.3 (1641.6 , 1850.1)** | **119919 (110359 , 129523)** | **1602.3 (1504.3 , 1706.6)** | **-8.1 (-12.3 , -3.6)** |
| **Kuwait** | **11102 (9849 , 12363)** | **995 (920.7 , 1075.8)** | **39087 (34844 , 43652)** | **1023.1 (939.3 , 1115.6)** | **2.8 (-1.4 , 6.9)** |
| **Lebanon** | **45238 (42396 , 48187)** | **1904.5 (1788.1 , 2030.3)** | **141237 (131990 , 150458)** | **2723.1 (2548.3 , 2901.7)** | **43 (38.1 , 47.8)** |
| **Libya** | **39402 (37040 , 41923)** | **1714.1 (1606.3 , 1832.5)** | **139857 (131289 , 147932)** | **2461 (2306.8 , 2612.1)** | **43.6 (38.1 , 49.8)** |
| **Morocco** | **223596 (208137 , 238935)** | **1365.6 (1274.9 , 1460.4)** | **611177 (573610 , 650326)** | **1896.7 (1782.4 , 2018.8)** | **38.9 (33.4 , 44.9)** |
| **Oman** | **8852 (7578 , 10134)** | **876.5 (784.4 , 968.7)** | **33346 (28850 , 38642)** | **1281.2 (1172.5 , 1395.8)** | **46.2 (39.6 , 53.6)** |
| **Palestine** | **17881 (16699 , 18965)** | **1707.3 (1607.6 , 1810.6)** | **44568 (40888 , 48163)** | **1552.7 (1454.1 , 1659.8)** | **-9.1 (-12.9 , -5.2)** |
| **Qatar** | **4075 (3757 , 4378)** | **1981.8 (1865.8 , 2101)** | **26415 (23313 , 29791)** | **1754.9 (1633.4 , 1886.3)** | **-11.4 (-15.2 , -7.7)** |
| **Saudi Arabia** | **101104 (91334 , 111224)** | **1381.3 (1285.4 , 1485)** | **434561 (396012 , 473597)** | **2053 (1918.1 , 2194.3)** | **48.6 (42.5 , 54.8)** |
| **Sudan** | **177280 (165885 , 189352)** | **1574 (1462.6 , 1697)** | **428128 (401529 , 455381)** | **1862.6 (1748 , 1989.1)** | **18.3 (14.1 , 23.6)** |
| **Syrian Arab Republic** | **108889 (102798 , 114953)** | **1651.1 (1557.4 , 1748.5)** | **249284 (233862 , 265450)** | **2017.3 (1903 , 2137.8)** | **22.2 (18.3 , 26.1)** |
| **Tunisia** | **82010 (75916 , 87875)** | **1543.5 (1445.8 , 1643.8)** | **249513 (233445 , 266354)** | **2048.5 (1916.8 , 2186.2)** | **32.7 (27.7 , 38.2)** |
| **Turkey** | **1007579 (965697 , 1047402)** | **2552.7 (2439.7 , 2668)** | **2885498 (2798099 , 2967100)** | **3287.1 (3187.4 , 3380.3)** | **28.8 (24.4 , 33.2)** |
| **United Arab Emirates** | **20338 (19552 , 21023)** | **2253 (2126 , 2379.7)** | **191068 (187010 , 194836)** | **2926.9 (2811.1 , 3046.4)** | **29.9 (24.4 , 36.4)** |
| **Yemen** | **110325 (103679 , 117954)** | **1732.8 (1612.1 , 1879.3)** | **342941 (323483 , 363074)** | **2065 (1932.8 , 2207)** | **19.2 (13.9 , 24)** |
