# Supplementary material for: The burden of chronic obstructive pulmonary disease and its attributable risk factors in the Middle East and North Africa region, 1990–2019
Source: Respir Res. 2022 Nov 19;23:319. doi: 10.1186/s12931-022-02242-z (PMC9675283; doi:10.1186/s12931-022-02242-z)
Supplement: Supplementary file 3 — Additional file 3: Table S3. Deaths of chronic obstructive pulmonary disease in 1990 and 2019 for both sexes and percentage change in age-standardised rates (ASRs) per 100,000 in the Middle East and North Africa region (generated from data available from http://ghdx.healthdata.org/gbd-results-tool). [file 12931_2022_2242_MOESM3_ESM.docx]

| **Table S3: Deaths from chronic obstructive pulmonary disease in 1990 and 2019 and the percentage change in the age-standardised rates (ASRs) per 100,000 in the Middle East and North Africa region**  **(Generated from data available from http://ghdx.healthdata.org/gbd-results-tool)** | | | | | |
| --- | --- | --- | --- | --- | --- |
|  | **1990** | | **2019** | | **Percentage change in ASRs per 100,000** |
|  | **No (95% UI)** | **ASRs per 100,000 (95% UI)** | **No (95% UI)** | **ASRs per 100,000 (95% UI)** |  |
| **North Africa and Middle East** | **43173 (37093 , 49741)** | **31.8 (26.9 , 36.7)** | **89920 (76785 , 101801)** | **26.1 (22.2 , 29.5)** | **-18 (-30.9 , -2.8)** |
| **Afghanistan** | **2877 (1624 , 3849)** | **48 (25.8 , 64.5)** | **3932 (2506 , 5144)** | **40.4 (24.2 , 52.6)** | **-15.7 (-36.2 , 7.8)** |
| **Algeria** | **2164 (1641 , 2768)** | **29.3 (22.7 , 37)** | **4905 (3836 , 6207)** | **20.5 (16 , 25.7)** | **-29.9 (-45.6 , -8.7)** |
| **Bahrain** | **58 (49 , 67)** | **60.5 (50.6 , 69.2)** | **111 (88 , 142)** | **28.9 (23.5 , 35.7)** | **-52.3 (-62.3 , -37.4)** |
| **Egypt** | **6881 (4992 , 8469)** | **28.9 (20.9 , 37.2)** | **14052 (9238 , 18627)** | **28.2 (18.5 , 36.8)** | **-2.6 (-27.6 , 30.7)** |
| **Iran (Islamic Republic of)** | **4230 (3709 , 5228)** | **23.4 (20.1 , 29.4)** | **12557 (10993 , 13621)** | **20.3 (17.7 , 22.1)** | **-13 (-30.5 , 1.7)** |
| **Iraq** | **785 (624 , 1000)** | **11.7 (9.3 , 14.7)** | **1711 (1343 , 2143)** | **9.8 (7.8 , 12.6)** | **-15.9 (-41 , 12.6)** |
| **Jordan** | **245 (193 , 294)** | **25.9 (20.7 , 31.2)** | **555 (450 , 680)** | **12.2 (9.9 , 15)** | **-53 (-64.7 , -37.4)** |
| **Kuwait** | **38 (32 , 43)** | **9.6 (8 , 11)** | **148 (117 , 182)** | **8.3 (6.5 , 10.3)** | **-12.7 (-30.8 , 8.4)** |
| **Lebanon** | **381 (294 , 484)** | **21.5 (16.7 , 27.3)** | **850 (631 , 1107)** | **16.8 (12.6 , 21.8)** | **-21.8 (-40.2 , 2)** |
| **Libya** | **286 (213 , 372)** | **17.8 (13.3 , 23.2)** | **760 (561 , 973)** | **17.6 (13.1 , 22.5)** | **-1.2 (-30.3 , 34.3)** |
| **Morocco** | **2346 (1718 , 3094)** | **21.7 (15.7 , 28.7)** | **5935 (4494 , 7387)** | **24.1 (18.6 , 29.7)** | **11.1 (-15.1 , 49.9)** |
| **Oman** | **154 (114 , 207)** | **34.2 (25.3 , 46.2)** | **202 (155 , 235)** | **23 (16.8 , 27.2)** | **-32.7 (-52.1 , -9.3)** |
| **Palestine** | **192 (146 , 273)** | **25.9 (19.8 , 36.8)** | **285 (234 , 368)** | **16.4 (13.4 , 21.4)** | **-36.5 (-61.4 , -14)** |
| **Qatar** | **15 (12 , 21)** | **26.6 (20.9 , 38)** | **49 (36 , 77)** | **20.2 (15.5 , 28.6)** | **-24.1 (-47.2 , 1.8)** |
| **Saudi Arabia** | **1433 (1073 , 2089)** | **33.6 (25.1 , 47.7)** | **2119 (1685 , 2576)** | **19.6 (15.9 , 23.4)** | **-41.4 (-61.9 , -20)** |
| **Sudan** | **2909 (1642 , 4231)** | **36.5 (20.6 , 53.2)** | **4390 (2883 , 6190)** | **28.7 (18.9 , 40.1)** | **-21.5 (-41.2 , 6.9)** |
| **Syrian Arab Republic** | **987 (734 , 1391)** | **21.9 (16 , 32.5)** | **1908 (1367 , 2781)** | **20.9 (15.4 , 30.4)** | **-4.8 (-30.6 , 33.8)** |
| **Tunisia** | **723 (568 , 976)** | **19.1 (14.9 , 25.4)** | **1831 (1325 , 2497)** | **16.8 (12.1 , 22.9)** | **-12 (-35.3 , 18.2)** |
| **Turkey** | **14764 (12162 , 16911)** | **48.4 (39.3 , 55.9)** | **29015 (19528 , 35902)** | **35.8 (24 , 44.4)** | **-26.1 (-46.4 , -4.1)** |
| **United Arab Emirates** | **141 (86 , 192)** | **41.9 (29.8 , 57.2)** | **1090 (653 , 1561)** | **31.2 (22.8 , 41.3)** | **-25.6 (-50.4 , 5.3)** |
| **Yemen** | **1538 (968 , 2198)** | **40.1 (25.8 , 58.2)** | **3423 (2591 , 4521)** | **33 (25 , 42.9)** | **-17.8 (-41.7 , 16)** |
