# Supplementary material for: The burden of chronic obstructive pulmonary disease and its attributable risk factors in the Middle East and North Africa region, 1990–2019
Source: Respir Res. 2022 Nov 19;23:319. doi: 10.1186/s12931-022-02242-z (PMC9675283; doi:10.1186/s12931-022-02242-z)
Supplement: Supplementary file 4 — Additional file 4: Table S4. DALYs due to chronic obstructive pulmonary disease in 1990 and 2019 for both sexes and percentage change in age-standardised rates (ASRs) per 100,000 in the Middle East and North Africa region (generated from data available from http://ghdx.healthdata.org/gbd-results-tool). [file 12931_2022_2242_MOESM4_ESM.docx]

| **Table S4: DALYs due to chronic obstructive pulmonary disease in 1990 and 2019 and the percentage change in the age-standardised rates (ASRs) per 100,000 in the Middle East and North Africa region**  **(Generated from data available from http://ghdx.healthdata.org/gbd-results-tool)** | | | | | |
| --- | --- | --- | --- | --- | --- |
|  | **1990** | | **2019** | | **Percentage change in ASRs per 100,000** |
|  | **No (95% UI)** | **ASRs per 100,000 (95% UI)** | **No (95% UI)** | **ASRs per 100,000 (95% UI)** |  |
| **North Africa and Middle East** | **1315162 (1164472 , 1461299)** | **736.1 (648.7 , 818.5)** | **2761836 (2436839 , 3062623)** | **649.1 (574.6 , 717.7)** | **-11.8 (-21.1 , -0.9)** |
| **Afghanistan** | **82231 (55748 , 107273)** | **1122.4 (745.1 , 1458.8)** | **137887 (100493 , 173594)** | **964.5 (681.8 , 1203.2)** | **-14.1 (-32.8 , 7.4)** |
| **Algeria** | **68470 (56013 , 82527)** | **579.1 (472.6 , 697.4)** | **148252 (124597 , 175872)** | **465.1 (390.8 , 548)** | **-19.7 (-34 , -1.9)** |
| **Bahrain** | **1707 (1503 , 1914)** | **1147.5 (996.2 , 1291)** | **4014 (3426 , 4797)** | **561.7 (484.6 , 662.1)** | **-51.1 (-59 , -39.5)** |
| **Egypt** | **230577 (184415 , 266825)** | **717.6 (560.9 , 836.7)** | **499452 (378041 , 615259)** | **776.4 (583.1 , 954.9)** | **8.2 (-12.2 , 34.3)** |
| **Iran (Islamic Republic of)** | **153371 (138121 , 176277)** | **555.9 (495 , 646.6)** | **372569 (338458 , 404911)** | **517.2 (471 , 560.8)** | **-7 (-20.6 , 3.2)** |
| **Iraq** | **29997 (25199 , 35564)** | **345.2 (289.3 , 412.9)** | **65299 (54269 , 76574)** | **265.2 (221.3 , 311.2)** | **-23.2 (-40.1 , -7)** |
| **Jordan** | **8223 (6955 , 9496)** | **597.2 (502.3 , 693.8)** | **22410 (19437 , 25914)** | **341.2 (297.1 , 392.9)** | **-42.9 (-53.4 , -30.7)** |
| **Kuwait** | **1850 (1594 , 2098)** | **245.1 (214.3 , 272.8)** | **6186 (5236 , 7195)** | **215.7 (185.7 , 249.7)** | **-12 (-23.7 , 1.9)** |
| **Lebanon** | **11965 (10017 , 14161)** | **541.9 (453.3 , 639.5)** | **27283 (22613 , 31817)** | **525.6 (436.8 , 612.6)** | **-3 (-18.7 , 13.8)** |
| **Libya** | **9586 (7969 , 11266)** | **467.4 (383.1 , 557.4)** | **28042 (23234 , 33105)** | **531.5 (440.8 , 630.6)** | **13.7 (-9.4 , 38.6)** |
| **Morocco** | **72430 (58330 , 87827)** | **509.3 (406.7 , 627.4)** | **172958 (141768 , 203952)** | **577.5 (476.2 , 680.6)** | **13.4 (-7 , 39)** |
| **Oman** | **4386 (3429 , 5769)** | **673.8 (517.4 , 879.1)** | **7193 (6122 , 8214)** | **453.9 (363.5 , 514.4)** | **-32.6 (-50.4 , -12.1)** |
| **Palestine** | **5537 (4473 , 7193)** | **605.1 (485.7 , 801.5)** | **9932 (8572 , 11625)** | **414.3 (359.2 , 492.5)** | **-31.5 (-52.4 , -14)** |
| **Qatar** | **774 (659 , 922)** | **593.7 (502.9 , 768.5)** | **3730 (3055 , 4557)** | **410.9 (345.6 , 522.7)** | **-30.8 (-45.7 , -15.2)** |
| **Saudi Arabia** | **39029 (30653 , 52588)** | **680.1 (532.6 , 941)** | **91982 (77925 , 107172)** | **508.2 (434.9 , 581.6)** | **-25.3 (-47.4 , -4.4)** |
| **Sudan** | **90846 (58643 , 130472)** | **849.2 (551.7 , 1164)** | **137492 (102426 , 179883)** | **682.3 (500.3 , 897.6)** | **-19.7 (-36.9 , 4)** |
| **Syrian Arab Republic** | **33833 (27944 , 40202)** | **562.7 (456.7 , 705.2)** | **64521 (52675 , 80806)** | **546.8 (448 , 686.7)** | **-2.8 (-23.5 , 25.1)** |
| **Tunisia** | **22554 (19137 , 27793)** | **453.2 (382.3 , 563.1)** | **54631 (44085 , 67863)** | **454.7 (367.8 , 563)** | **0.3 (-19.1 , 23.8)** |
| **Turkey** | **389298 (338506 , 434352)** | **1099 (953.2 , 1230.2)** | **733647 (575036 , 858889)** | **855.2 (667.5 , 1000.4)** | **-22.2 (-36.7 , -6.1)** |
| **United Arab Emirates** | **7028 (4614 , 9077)** | **1001.4 (738.8 , 1279.3)** | **59161 (39649 , 78876)** | **886.1 (698.8 , 1101.3)** | **-11.5 (-35.9 , 19.3)** |
| **Yemen** | **50586 (35978 , 68098)** | **919.1 (641.4 , 1247.8)** | **112389 (90488 , 140804)** | **785.1 (634.1 , 976)** | **-14.6 (-36.3 , 14.5)** |
