# Supplementary figures and images for: The burden of chronic obstructive pulmonary disease and its attributable risk factors in the Middle East and North Africa region, 1990–2019
Source: Respir Res. 2022 Nov 19;23:319. doi: 10.1186/s12931-022-02242-z (PMC9675283; doi:10.1186/s12931-022-02242-z)

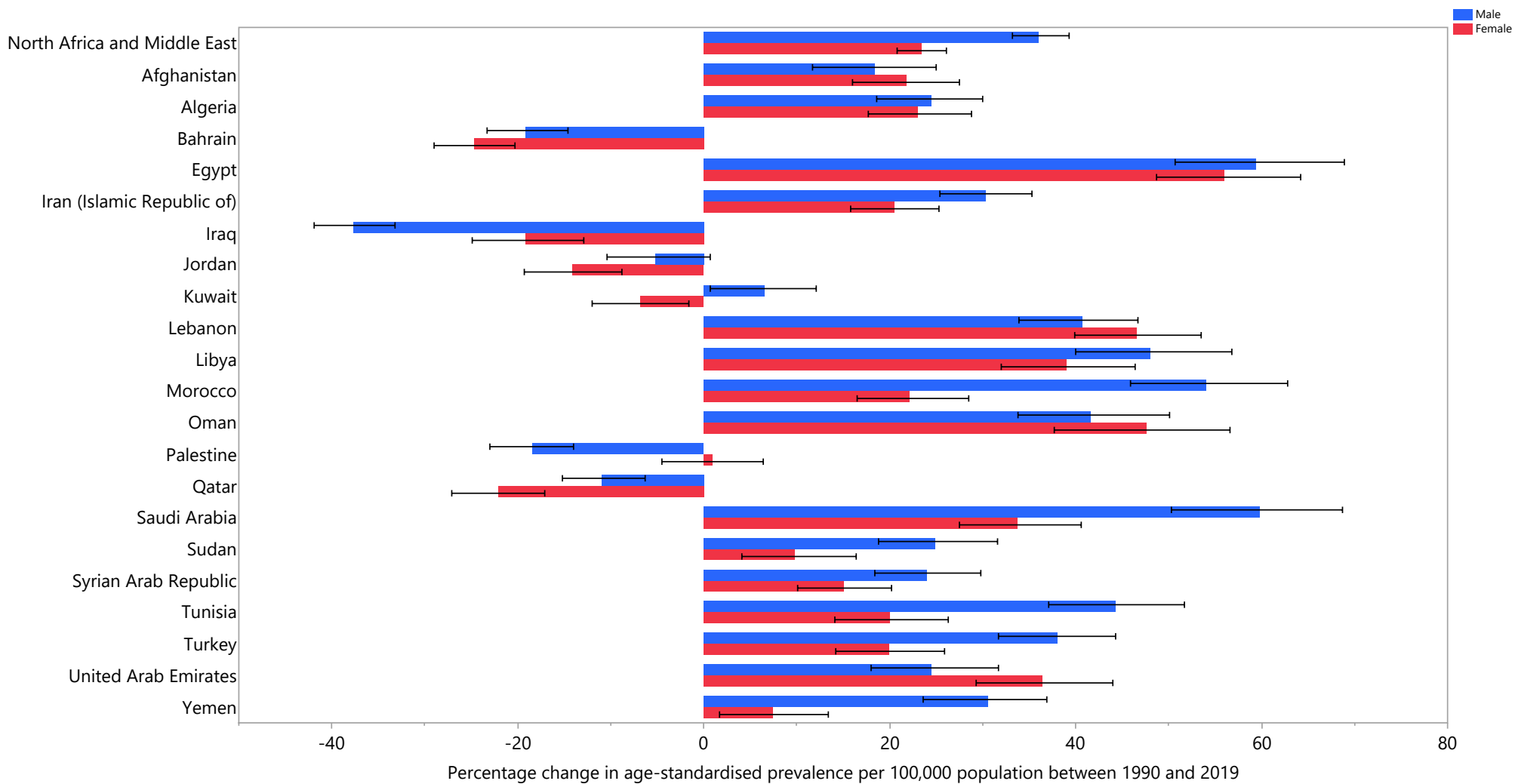

Supplement: Supplementary file 5 — Additional file 5: Figure S1. The percentage change in the age-standardised point prevalence of chronic obstructive pulmonary disease in the Middle East and North Africa region from 1990 to 2019, by sex and country (generated from data available from http://ghdx.healthdata.org/gbd-results-tool). [file 12931_2022_2242_MOESM5_ESM.pdf]

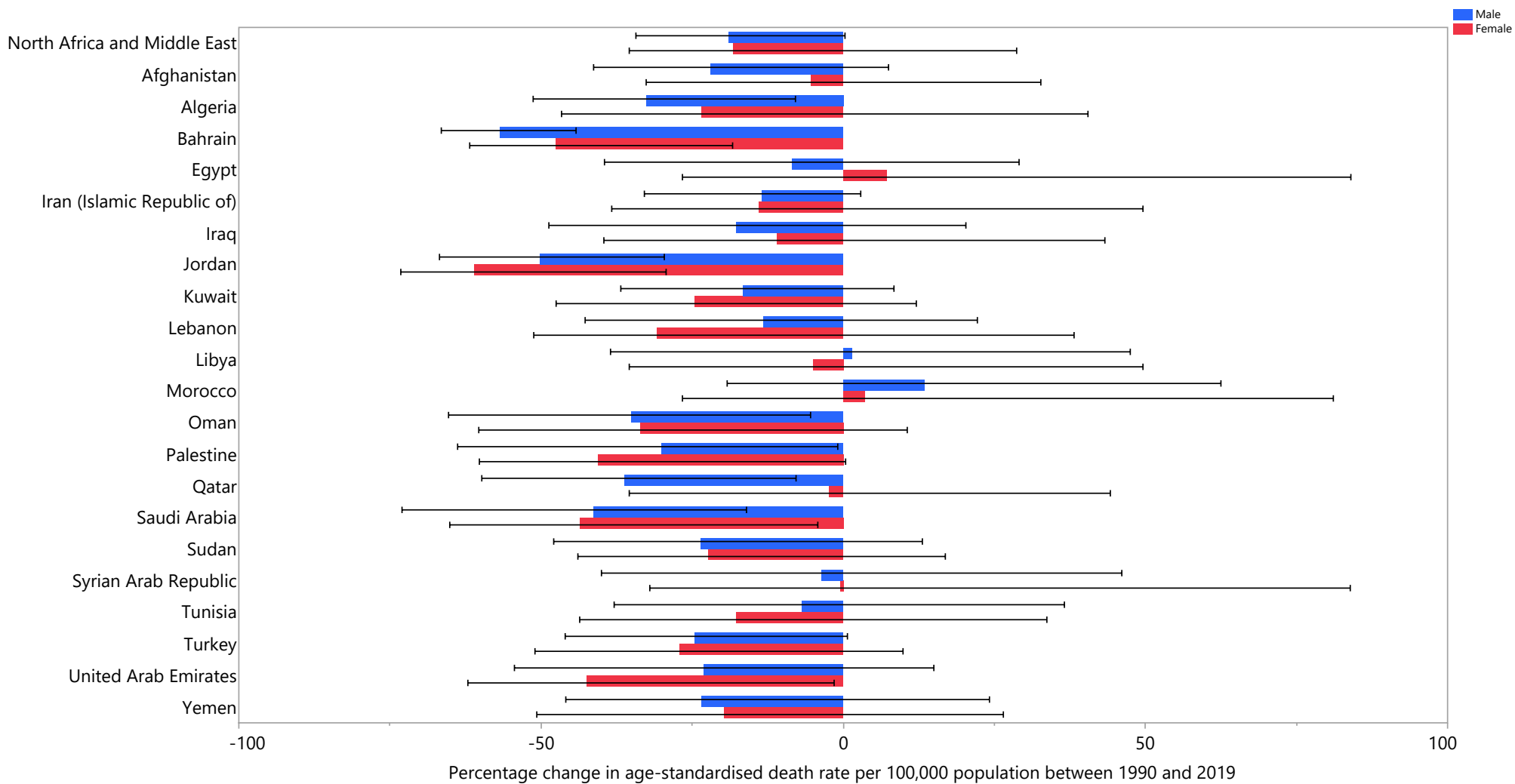

Supplement: Supplementary file 6 — Additional file 6: Figure S2. The percentage change in the age-standardised death of chronic obstructive pulmonary disease in the Middle East and North Africa region from 1990 to 2019, by sex and country (generated from data available from http://ghdx.healthdata.org/gbd-results-tool). [file 12931_2022_2242_MOESM6_ESM.pdf]

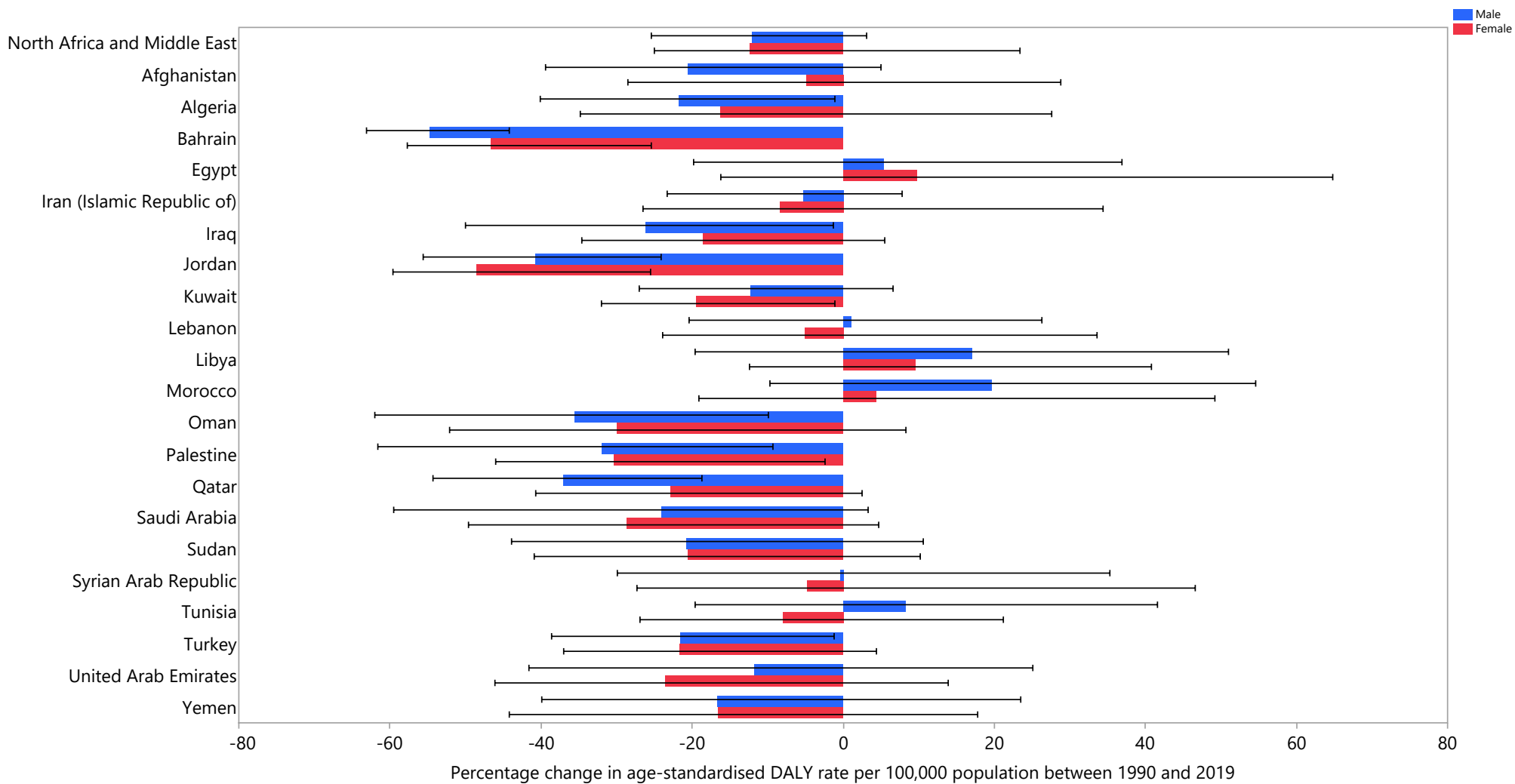

Supplement: Supplementary file 7 — Additional file 7: Figure S3. The percentage change in the age-standardised DALYs of chronic obstructive pulmonary disease in the Middle East and North Africa region from 1990 to 2019, by sex and country. DALY = disability-adjusted-life-years (generated from data available from http://ghdx.healthdata.org/gbd-results-tool). [file 12931_2022_2242_MOESM7_ESM.pdf]

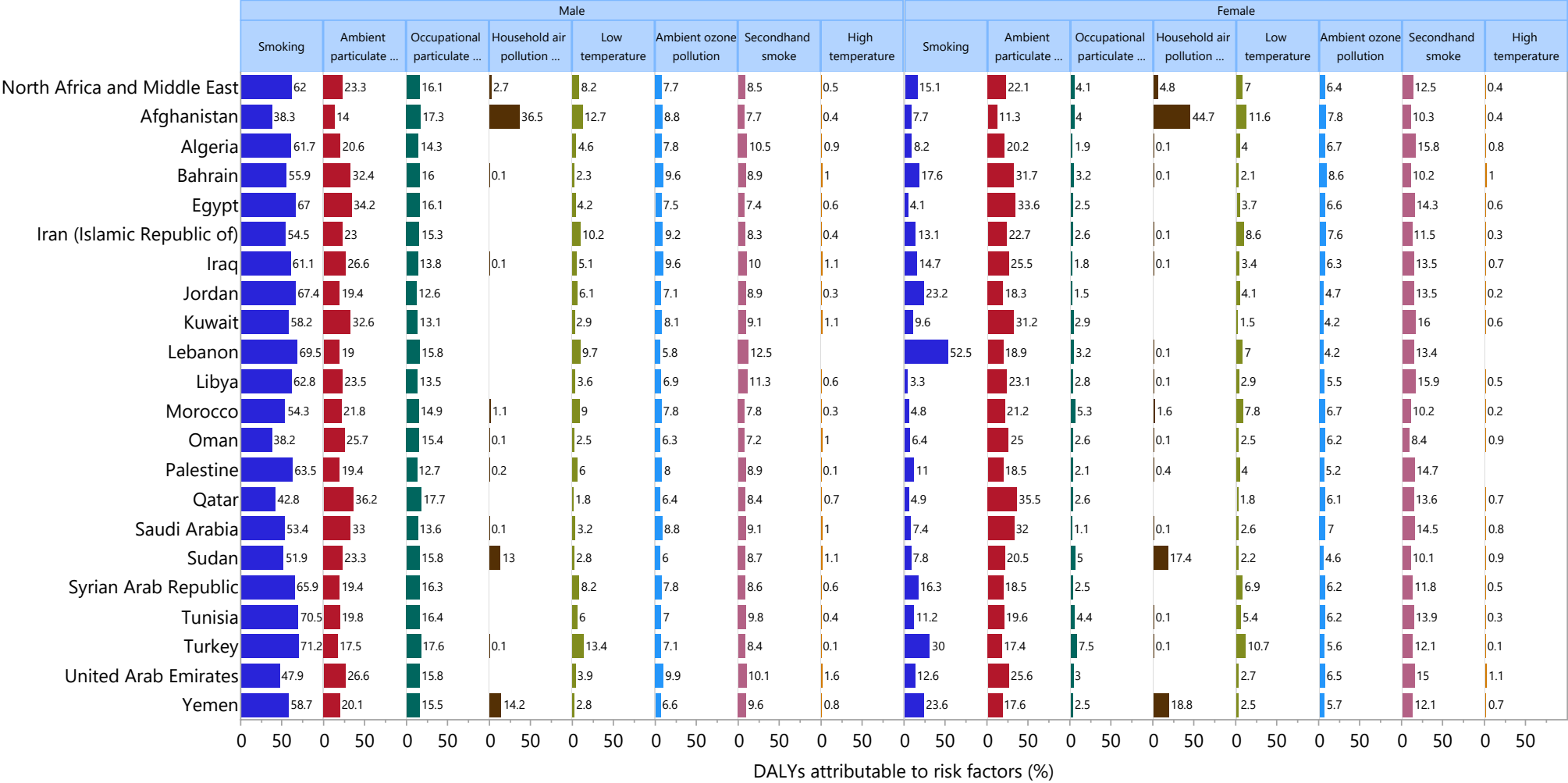

Supplement: Supplementary file 8 — Additional file 8: Figure S4. Percentage of DALYs due to chronic obstructive pulmonary disease attributable to risk factors for the Middle East and North Africa countries, by sex, in 2019. DALY = disability-adjusted-life-years (generated from data available from http://ghdx.healthdata.org/gbd-results-tool). [file 12931_2022_2242_MOESM8_ESM.pdf]
